# Case report of familial sudden cardiac death caused by a DSG2 p.F531C mutation as genetic background when carrying with heterozygous KCNE5 p.D92E/E93X mutation

**DOI:** 10.1186/s12881-018-0580-2

**Published:** 2018-08-21

**Authors:** Yubi Lin, Jiana Huang, Siqi He, Ruiling Feng, ZhiAn Zhong, Yang Liu, Weitao Ye, Xin Li, Hongtao Liao, Hongwen Fei, Fang Rao, Zhixin Shan, Chunyu Deng, Xianzhang Zhan, Yumei Xue, Hui Liu, Bin Zhang, Kejian Wang, Qianhuan Zhang, Shulin Wu, Xiufang Lin

**Affiliations:** 1 Department of Cardiology and Cardiovascular Intervention, Interventional Medical Center, The Fifth Affiliated Hospital of Sun Yat-sen University, Zhuhai, 519000 People’s Republic of China; 2 Guangdong Cardiovascular Institute, Guangdong Academy of Medical Sciences, Guangdong General Hospital, Guangdong Provincial Key Laboratory of Clinical Pharmacology, Affiliated to Medical school of South China University of Technology, Guangzhou, 510080/520006 People’s Republic of China; 3 Jinan University, Guangzhou, 510630 China; 4 Department of Radiology, Guangdong General Hospital, Guangdong Academy of Medical Sciences, Guangzhou, 510080 People’s Republic of China; 5 Department of Echocardiography, Guangdong General Hospital, Guangdong Academy of Medical Sciences, Guangzhou, 510080 People’s Republic of China; 6 Lin He’s Academician Workstation of New Medicine and Clinical Translation at The Third Affiliated Hospital, Guangzhou Medical University, Guangzhou, 510150 People’s Republic of China

**Keywords:** Sudden cardiac death, Arrhythmogenic right ventricular cardiomyopathy/dysplasia, Ventricular tachycardia, Electrical storm, Genetics

## Abstract

**Background:**

Sudden cardiac death (SCD) induced by malignant ventricular tachycardia (MVT) among young adults with right ventricular cardiomyopathy/dysplasia (ARVC/D) is a devastating event. Parts of ARVC/D patients have a mutation in genes encoding components of cardiac desmosomes, such as desmoglein-2 (DSG2), plakophilin-2 and desmoplakin.

**Case presentation:**

Here we report a potentially pathogenic mutation in the *DSG2* gene, which was identified in a family with ARVC/D using Whole Exome Sequencing (WES) and Sanger Sequencing. In all, Patient III:1 with ARVC/D carried the compound heterozygous mutations of DSG2 p.F531C and KCNE5 p.D92E/E93X, which were both inherited from her mother (II:2), who died of SCD. Carriers of *DSG2p.F531C* showed various phenotypes, such as ARVC/D, SCD, MVT and dilated cardiomyopathy. For III:1, there were significant low-voltage regions in the inferior-apical, inferior-lateral wall of the right ventricular epicardium and outflow tracts of the right ventricle. Under the guidance of a three-dimensional mapping system, MVT was successfully ablated with an epicardial–endocardial approach targeting for late, double or fragmental potentials after implantable cardioverter-defibrillator (ICD) electrical storms. No VT recurrence was observed during the one year of follow-up.

**Conclusions:**

When coexisting with heterozygous KCNE5 p.D92E/E93X, heterozygous *DSG2 p.F531C* as a genetic background was found to predispose to ARVC/D, SCD and MVT, which were successfully ablated using an epicardial–endocardial approach.

**Electronic supplementary material:**

The online version of this article (10.1186/s12881-018-0580-2) contains supplementary material, which is available to authorized users.

## Background

Sudden cardiac death (SCD) is a major public health problem accounting for 15%–20% of all deaths [1]. Common causes of SCD include cardiomyopathies and ion channelopathies in the young population aged < 35 years [2]. SCD can result in death among patients with arrhythmogenic right ventricular cardiomyopathy/dysplasia (ARVC/D), sometimes with early signs of malignant ventricular tachycardia (MVT) of hemodynamic compromise [3]. ARVC/D is a genetically determined cardiomyopathy characterized by fibro-fatty replacement of the right ventricle or in some cases the left ventricle. Less than 60% of ARVC/D patients have a mutation in genes encoding components of cardiac desmosomes, such as plakophilin-2, desmoglein-2 (DSG2) and desmoplakin [4]. Some patients implanted with implantable cardioverter-defibrillator (ICD) still suffer from electrical storms or SCD due to repeated MVT. This is associated with a history of a cardiac arrest or MVT, younger age, unexplained syncope, presence of non-sustained VT and other risk factors [5, 6]. In this study, we examined a Chinese Han family with ARVC/D, dilated cardiomyopathy (DCM), MVT and a history of SCD. The genetic background of the family members was explored by whole exome sequencing (WES). Meanwhile, MVT was mapped in one patient under the guidance of a three-dimensional mapping system.

## Case presentation

**Fig. 1:**
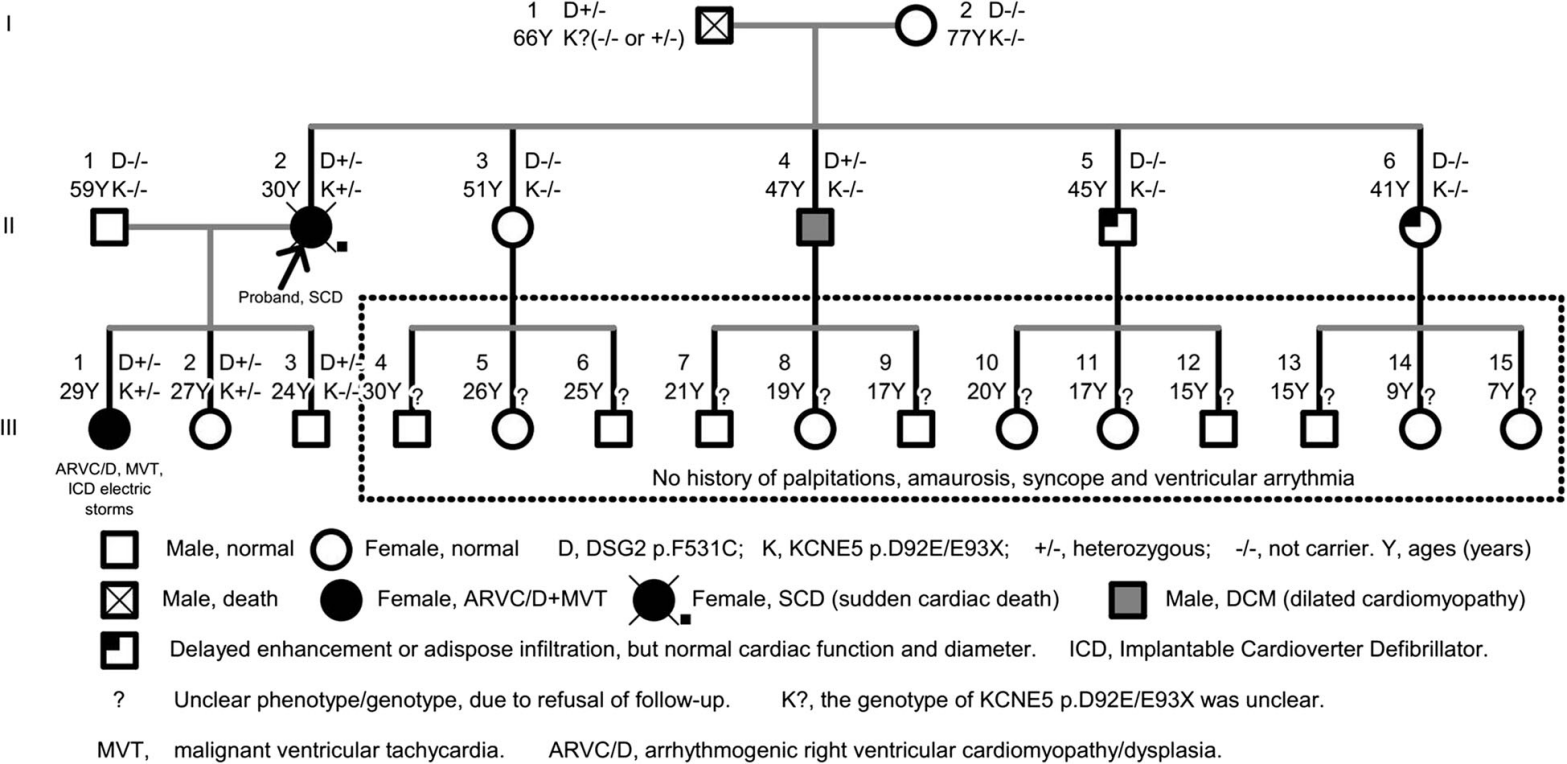
Pedigree Charts

This study was approved by the Guangdong Medical Institutional Review Board and Medical Ethics Committees [No.GDREC2016001H (R1)], and all participants gave informed consent. The CARE guidelines were followed as well. Family members III:4–15 were not followed up due to the absence of clinical symptoms or because they were under the age of 18 (Fig. 1). Detailed clinical information was obtained, including family history, age of presentation, initial symptoms of VT and cardiomyopathy, physical examination, electrocardiograms (ECGs), echocardiograms and cardiac computed tomography (CT) /magnetic resonance image (MRI) based on their informed consent. In addition, Holter monitoring, electrophysiological examination and three-dimensional cardiac reconstruction (guided by the CARTO system) were performed in patient III: 1. Familial cardiac disease was diagnosed according to the WHO1995 diagnostic criteria and Revised (2010) Task Force Criteria for Diagnosis of ARVC/D [7]. Individuals without common diseases or minor cardiac or skeletal muscle abnormalities were classified as healthy. In this family, patient III:1 was diagnosed with ARVC/D and MVT and thus elected to WES.

### Whole exome sequencing

Genomic DNA samples of the family members were isolated from peripheral blood using a standard DNA extraction protocol. The isolated genomic DNA of III:1 was then fragmented into 150–200 bp and subjected to DNA library preparation using established Illumina paired-end protocols. Adaptor-ligated libraries were amplified via PCR. A portion of each library was used to create an equimolar pool. Each pool was amplified to enrich for targets to be sequenced by the Agilent SureSelectXT Target Enrichment System (Agilent Technologies Inc., Santa Clara, CA, USA). Whole exome capture was performed with the Agilent SureSelectXT Human All Exon 50 Mb Kit (Agilent Technologies Inc.) following the manufacturer’s protocol. The exome-enriched libraries were sequenced with the Illumina HiSeq2000 platform (Illumina, San Diego, CA, USA) according to the manufacturer’s instuctions, and 100 bp paired-end sequencing reads were generated. Each sample was sequenced per lane to obtain an average theoretical depth of 100 × .

### Read mapping, variant detection and functional annotation

Raw reads of WES for III:1 were collected for quality control, in which low quality reads were filtered and 3′/5′ adapters were trimmed using the Trim Galore program. Clean reads were aligned to the human reference genome (University of California Santa Cruz, UCSC build hg19) using the Burrows-Wheeler Aligner (BWA) program. The quality scores were recalibrated, and reads were realigned to the reference genome using the Genome Analysis Toolkit (GATK) software package. Following the exclusion of duplicate reads, insertion-deletions (InDels) and single-nucleotide polymorphisms (SNPs) were called using the GATK or Sequence Alignment/Map tools (SAM tools).

SNPs and InDels were annotated using a pipeline in which all insertion and deletion variants occurring incoding regions were considered damaging, and nonsynonymous SNPs were predicted by SIFT (http://sift.jcvi.org/www/) [8] and, PolyPhen-2 (Polymorphism Phenotyping v2, http://genetics.bwh.harvard.edu/pph2/) [9] .The variants of III:1 in approximately 200 genes (showed in Additional file 1) predisposing to hereditary cardiomyopathies and arrhythmias were screened as candidate genes, and the filtering criteria for variant inclusion were as follows: (1) same variants in the WES data; (2) missense, nonsense and InDel variants; and (3) SNPs with a minor allele frequency not more than 0.01 according to the NCBI-SNP database [10, 11].

### Sanger sequencing for candidate genes

DNA sequences of the candidate genes were obtained from Genbank, and the primers were designed with Primer Premier 5.0. The protein-coding regions of these genes were amplified from genomic DNA. Amplification products were purified using the MinElute PCR Purification Kit (Qiagen) and were sequenced directly with the BigDye terminator method (Applied Biosystems, Foster City, CA, USA) on a capillary autosequencer (ABI Prism 3100) using the sequencing primers shown in Table 1. Three dimensional structure analyses were conducted with the SWISS-Model Server using alignment mode.

**Table 1:** Candidate gene primers

Genes	Forward primer	Reverse primer	Length	Annealing temperature
OBSCN	GTGGGACGAAGCGAGGGTA	GAGATGGGGCAGGATGAAGG	619 bp	60.8 °C
ALDH1A2	AGTTAGCCTTTGGATGATGTTA	TTTGCTGCTCTGCTGTTTG	376 bp	50.1 °C
ABCA3	GTAGTCCCCGTGGTCCTCTG	GCTCTGGCTGCTGACCTGA	362 bp	59.0 °C
COL3A1	AGGGAAGTCAAGGAGAAAGT	CACACATACAACCATAACCAAT	278 bp	50.5 °C
DSG2	AGGGAATTCAAACTATGTCTGT	AACTACTACGATTGTGGTGCT	596 bp	55.0 °C
SYNE1	GTTTGATTGTCTTTTTTGTT	GAATAGTCTCTTCTTATCTGGA	463 bp	51.1 °C
KCNE5	GCGGGAGTGAGGGAATAAGG	GCAGGGGTGAAGAGGGAGAAA	832 bp	62 °C

### Electroanatomic mapping

The epicardial access was secured through a subxiphoid puncture. The decision to proceed to epicardial mapping and ablation was left to the operator’s discretion. Electroanatomic mapping was performed during sinus rhythm using CARTO-V3 (Biosense-Webster, Diamond Bar, CA, USA), for III:1. Mapping was performed with a multipolar high-density mapping catheter (PentaRayNav, Biosense Webster). We defined a peak-to-peak bipolar amplitude of < 1.5 mV in the endocardium and < 1.0 mV in the epicardium as the bipolar low-voltage or scar zones [12]. The local abnormal ventricular activities were labeled in the mapping model.

### Follow-up

III:1 was followed up for one year with ICD interrogation, in case of the recurrence of sustained VT or appropriate ICD therapies.

### Familial characteristics

**Fig. 2:**
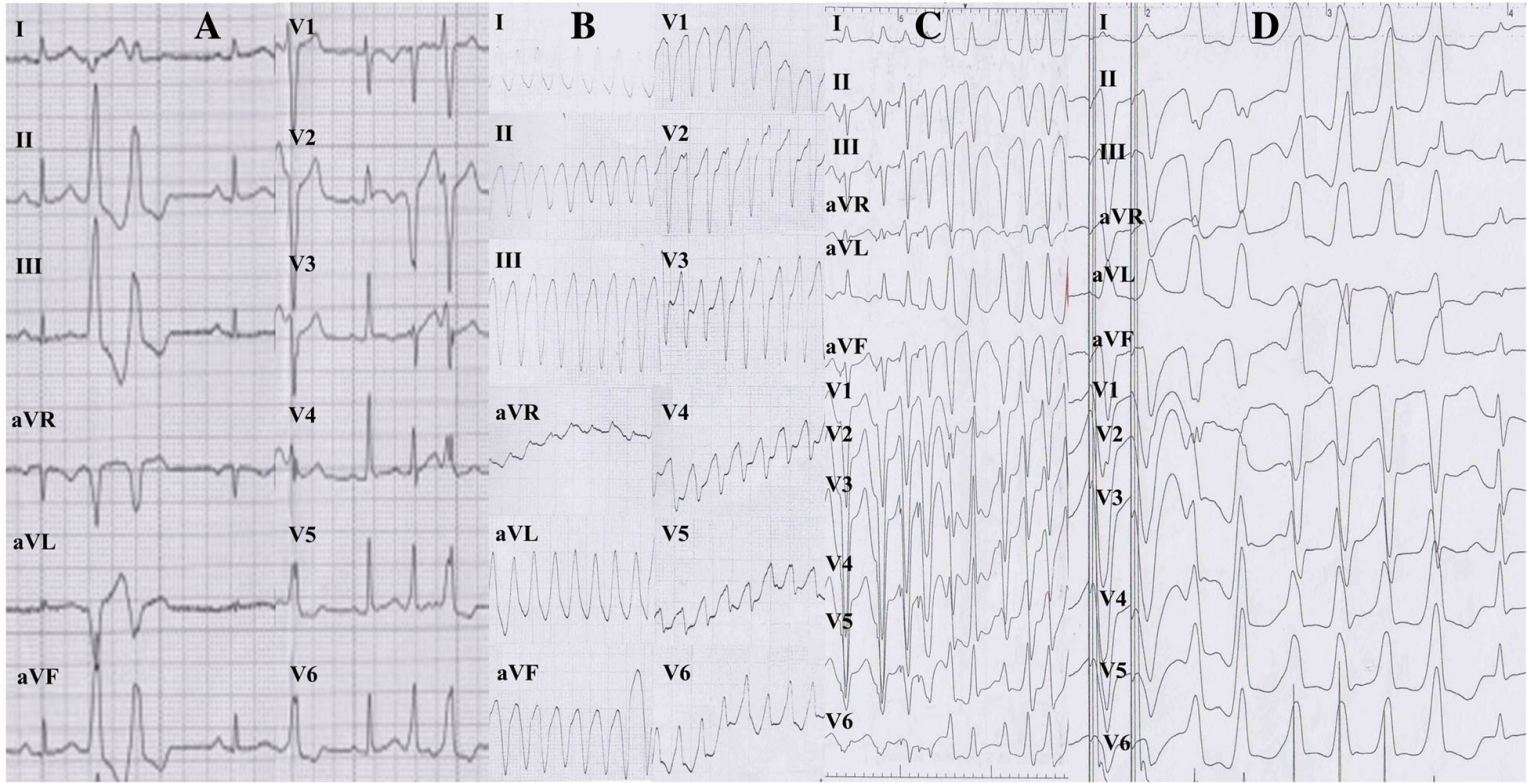
ECGs characteristics of III:1 patient. **a** Inversion of T wave (V_1_-V_3_ leads) in sinus rhythm; Pairs of ventricular premature beat origin from right ventricular tract; cardiac counterclockwise transposition. **b** Clinical episode of persistent and malignant ventricular tachycardia origin from the inflow-free wall of right ventricle. **c**–**d** Ventricular programed stimulation induced short episode of ventricular tachycardia origin from the inflow-free wall (**c**) and outflow tract of right ventricle (**d**), during first ablation in the other hospital

**Fig. 3:**
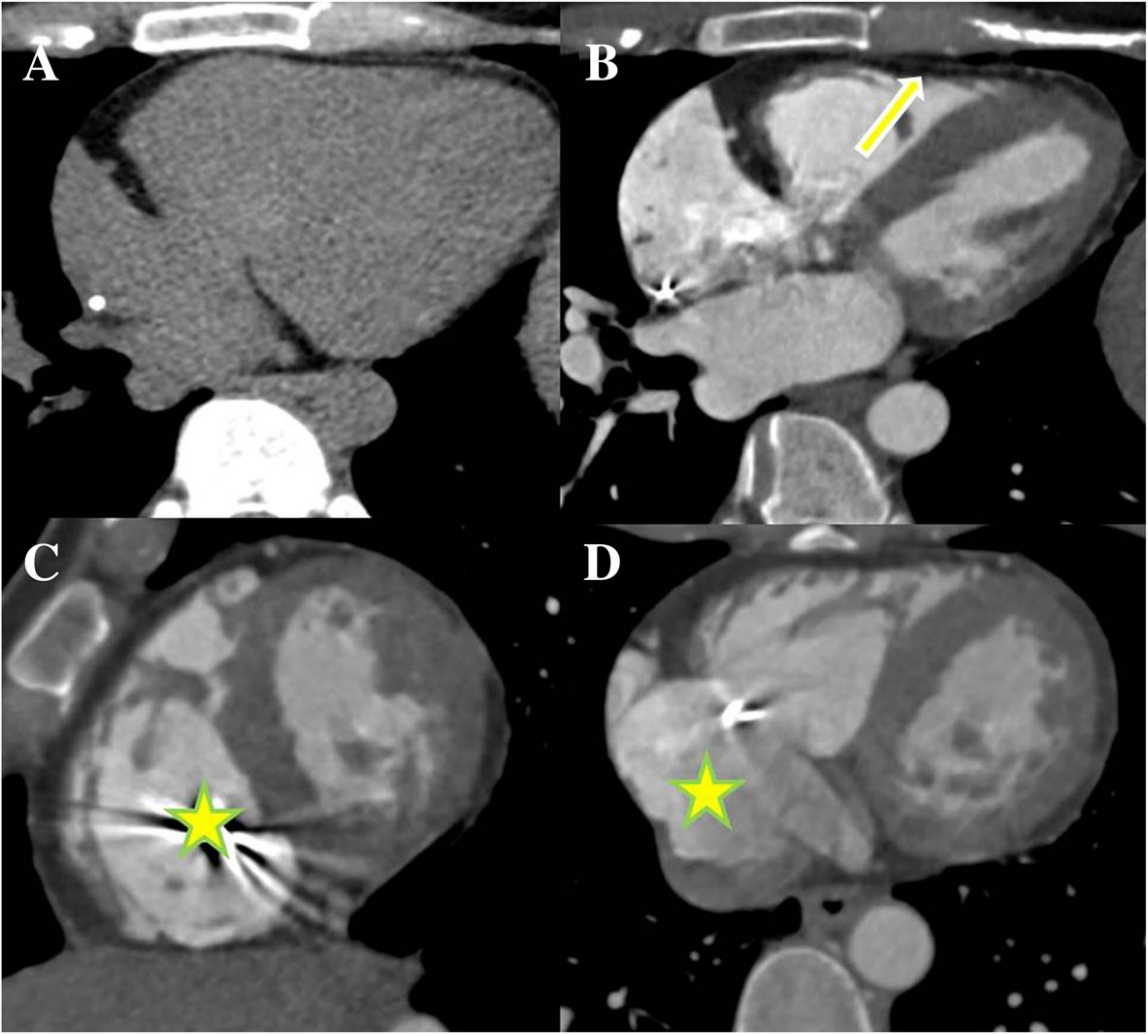
Cardiac CT images of III:1 patient. Four-chamber view of non-enhanced image (**a**) and enhanced image (**b**). Thinning of the anterior wall of the right ventricle with extensive fatty infiltration (arrow) could be noted. The short-axis view (**c**) demonstrated dilation of the right ventricle (star). Enlargement of the right atrium (stars) was shown on the axial view (**d**)

The proband, II:2 (female, 30 years old), had experienced eight episodes of unexplained syncope, but had never been medically examined or treated. Unexplained sudden death occurred while she was doing housework in the kitchen. Another affected patient, III:1 (female, 25 years old, offspring of patient II:2), experienced repeated sudden heart palpitations and syncope for three months, with loss of consciousness, foaming at the mouth and tic of limbs that lasted for approximately 30 s and spontaneously released without drug therapy and any medical care. The similar symptoms above were recurrent without any reason or medical therapy before, and her emergency ECG showed with persistent VT derived from the inflow-free wall of right ventricle (Fig. 2), which was restored by intravenous injection of Lidocaine at 15th September of 2013. Due to firstly unsuccessful catheter ablation targeting for VT after the electrophysiological examination, guided by the CARTO system in another hospital, an ICD was implanted. Although III:1 administered with oralβ-blocker (metoprolol sustained release tables, 37.5 mg Qd), repeated ICD electric storms had reoccurred 20 times during the one year follow-up. III:1 came for further therapy. The cardiac CT examination (Fig. 3) of III:1 indicated the right atrial and ventricular extension, and fat infiltration in the right ventricle, which was diagnosed with ARVC/D. Cardiac magnetic resonance imaging (CMRI) of II:4 showed DCM, characterized as linear and delayed enhancement in the left ventricular septum, myocardial thinning in the apex of left ventricle, moderate enlargement of left ventricle and moderate-severe decrease of systolic function and a mild decrease in systolic function of the right ventricle with a normal diameter. Delayed enhancement in the epicardial base and adipose infiltration in the apex of left ventricle were demonstrated in II:5 and II:6 respectively without abnormal cardiac diameter and systolic motion in both ventricles (Fig. 1). III:2 without clinical syndrome and arrhythmias demonstrated by 24-h Holter recording, had normal characteristics of ECG and CMRI, and thus refused further electrophysiological examination. III:3 refused further cardiac MRI due to no clinical syndrome and normal characteristics of ECG and 24-h Holter recording in the first follow-up. The ECG of III:1-3 had no characteristics of Brugada, long QT or short QT syndrome. Patient I:1 suffered from hypertension and experienced two cerebral strokes. He was bedridden for 2 years before dying at the age of 66. Patient I:2 had hypertension, type 2 diabetes mellitus and myocardial infarction. Other family members (III:4–15) had no abnormal clinical symptoms or VT in 24-h Holter monitoring, and declined further examination.

### Genetic screening for potentially pathogenic mutations

**Fig. 4:**
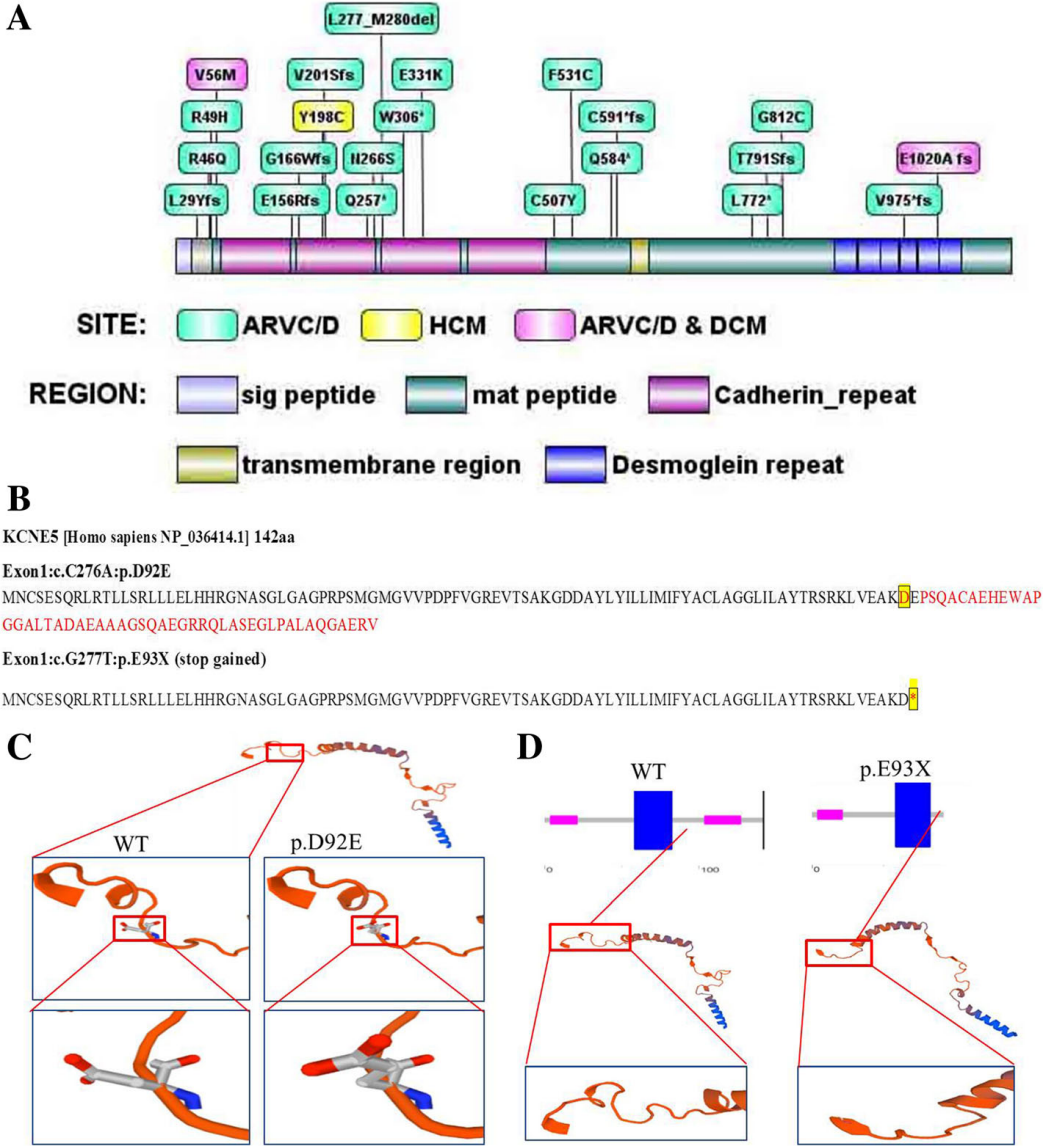
Pathogenic mutations in the DSG2 protein and structure of KCNE5 mutations. **a** secondary structure of DSG2 protein (NP_001934.2), which consists of 1118 amino acids. The pathogenic mutation related to ARVC/D, hypertrophic cardiomyopathy (HCM) and dilated cardiomyopathy (DCM) were displayed according to PubMed ClinVar and recent reports from PubMed. **b**-**d** primary (**b**) and tertiary (**c**–**d**) structure changes of KCNE5 p.D92E/E93X mutation and wildtype of KCNE5, constructed by Swiss-model. *, stop-gain

In this family (Fig. 1), a set of candidate genes associated with cardiomyopathies and arrhythmias (as shown in the Additional file 1) were screened using the WES data of III:1. The results showed that III:1 carried the heterozygous mutations in *DSG2 (p.F531C)*, *KCNE5 (p.D92E/E93X)*, *ALDH1A2 (p.E50G)* and *SYNE1 (p.Q8498R)*, but these mutations were not present in her father (II:1) (Table 2). Furthermore, *DSG2 (p.F531C)* was also detected in III:2, III:3, but negative for II:1. Therefore, we inferred that *DSG2 (p.F531C)* of III:1 was likely inherited from II:2 who died of SCD. In addition, *DSG2 (p.F531C)* was carried by II:2 and II:4, but negative for I:2, thus It was speculated that *DSG2 (p.F531C)* was heterozygous in I:1 although lack of DNA sample and analysis of sanger sequencing for I:1 who had been dead for a long time bwefore our study. Prediction with SIFT/Polyphen-2 revealed that *DSG2 p.F531C* may be a deleterious mutation that is not found in the East Asian population of the 1000 Genomes Project (2015 release version). *DSG2* consists of 1118 amino acids, including extracellular (four Cadherin-repeats), transmembrane and intracellular domains. The pathogenic mutation related to ARVC/D, hypertrophic cardiomyopathy and DCM were displayed according to NCBI ClinVar database and recent reports from PubMed. The *DSG2 p.F531C* is located between the fourth cadherin structure and the transmembrane region (Fig. 4a).

**Table 2:** Potential and pathogenic genes identified by whole exome sequencing and predisposed to cardiomyopathies and arrhythmias

Chr	Start	Genes	AA-Change	1000G	SNP	SIFT	PP	LRT	MT	III:1	II:1
chr1	228,504,652	OBSCN	NM_001098623:exon51:c.G13528A:p.D4510N	–	–	D(0.011)	D(0.967)	N(0.002)	D(1)	±	±
chr15	58,306,448	ALDH1A2	NM_003888:exon2:c.A149G:p.E50G	0.002	rs34266719	D(0.013)	B(0.062)	D(0)	D(1)	±	–
chr16	2,329,121	ABCA3	NM_001089:exon29:c.G4370A:p.R1457Q	0.001	rs201226715	T(0.063)	B(0.327)	D(0)	D(1)	±	±
chr18	29,116,333	DSG2	NM_001943:exon11:c.T1592G:p.F531C	–	rs200484060	D(0)	D(0.998)	D(0)	D(0.999)	±	–
chr2	189,861,158	COL3A1	NM_000090:exon24:c.C1697T:p.P566L	0.006	rs150543864	T(0.101)	D(0.916)	N(0.003)	D(1)	±	±
chr6	152,457,775	SYNE1	NM_033071:exon141:c.A25493G:p.Q8498R	0.001	rs529921934	T(0.705)	D(0.974)	D(0)	D(1)	–	–
chrX	108,867,973	KCNE5	NM_012282:exon1:c.G277 T:p.E93X	0.0026	rs61729624	–	–	N(0.025)	D(1)	±	–
chrX	108,867,974	KCNE5	NM_012282:exon1:c.C276A:p.D92E	0.0026	rs200723915	T(0.254)	B(0.005)	N(0.003)	N(0.99)	±	–

*Chr* chromosome, *AAChange* amino acid change, *1000G* 1000genomes 2015, *SNP* single nucleoside polymorphism, *PP* polyphen-2, *MT* Mutation-Taster. *D* damaging, *B* benign, *T* tolerated, *N* nature; ± heterozygous carrier; −, non-carrier

According to the 1000 Genomes Project (2015 release version), the minor allele frequency of *KCNE5 (p.D92E/E93X)* was 0.0026 in this population. *KCNE5 (p.D92E/E93X)* was detected in III:1 and III:2, but negative for III:3 and II:1, which suggested that *KCNE5 (p.D92E/E93X)* was heterozygous in II:2 who died of SCD*.* Based on the familial and detailed analysis of *KCNE5 (p.D92E/E93X),* which was negative for the other family members including II:3-6 and I:2, we still couldn’t speculate what the exact genotype of *KCNE5(p.D92E/E93X)* was for I:1. *KCNE5 (p.D92E/E93X)* it was possibly homologous negative or heterozygous carried by I:1 (Fig. 1), but inclined to the former. The 51 to 94 amino acids of *KCNE5* protein form the slow voltage-gated potassium channel (or ISK Channel); amino acids 61 to 81, 82 to 103 and 104 to 126 are located in the transmembrane region, an unknown region and a low complexity region respectively (Fig. 4b). Therefore, *KCNE5 p.E93X* results in a shortened sequence that truncates the protein from 93 to 142 amino acids (Fig. 4c–d).

The minor allele frequency of *ALDH1A2 p.E50G* (rs34266719), which was detected in III:1 and III:2, was 0.002 in the 1000 Genomes Project. *ALDH1A2* is an enzyme that catalyzes the synthesis of retinoic acid from retinaldehyde and regulates tissue development. The *SYNE1 p.Q8498R* (rs529921934) mutation from WES was validated as a false positive by Sanger sequencing. The mutations *OBSCN p.D4510N*, *ABCA3 p.R1457Q* and *COL3A1 p.P566L* were inherited from the patient’s father (II:1), who did not have cardiac disease and any adverse events.

### Substrate mapping and catheter ablation

**Fig. 5:**
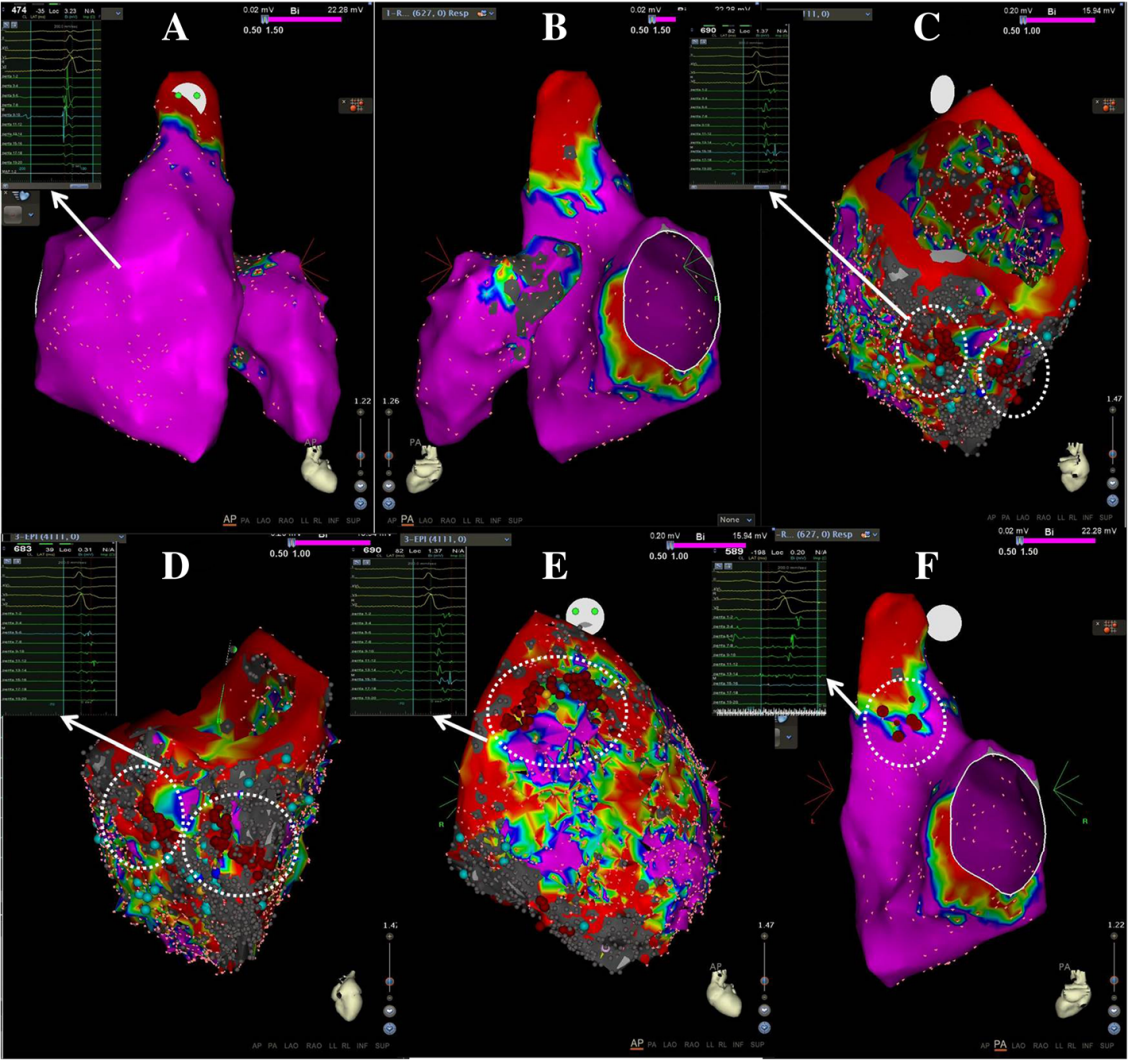
Electrical and anatomical mapping with guidance of CARTO system. **a** Endocardial mapping of both ventricles, showed in anterior-posterior position. **b** Endocardial mapping of both ventricles, showed in posterior–anterior positions. **c**–**d** Epicardial mapping indicated that there were obvious low-voltage regions or scars with significant late, double or fragmented potentials, around the tricuspid annulus, in inferior-lateral-apex and inferior-apex walls (**c**–**d**), and outflow tract (**e**) of right ventricle. Line and lamellar ablation was performed across the scars (red points). F, in the mid-septum of endocardium in the right ventricle, the pacing mapping suggested potential targets, as ECG characteristics of pacing similar to that of short episodes of clinical VT. This region was mapped with significant late potentials and ablated (red points)

For III:1, short episode of VTs originating from the right ventricular inflow and outflow tracts were observed during the electrophysiological examination. Under the guidance of the CARTO system and mapping (Fig. 5), we found no obvious region of low voltage, scars or abnormal potential in the endocardium of both ventricles. We continued mapping in the epicardium, and found slow conductions in the apical, basal and outflow regions, surrounding the tricuspid annulus (6–9 counterclockwise) and the inferior-lateral wall of right ventricle. The slow conductions were characterized by narrow and late double potentials or fragmental potentials for potential targets. Next, linear or lamellar ablation was performed in these areas. At the same time, the median septum of the right ventricular outflow tract in the endocardium was conducted by pacing mapping, and then ablation was performed after identifying potential targets. There was no VT induction after intravenous infusion of isoproterenol. During the one-year of follow-up after ablation, VT didn’t occur during the oral administration of β-blocker (metoprolol sustained release tablets, 47.5 mg every two days).

## Discussion and conclusions

We detected the compound heterozygous mutations of *DSG2 p.F531C* and *KCNE5 p.D92E/E93X* as the important risks for the development of ARVC/D, DCM, MVT and SCD. *DSG2 p.F531C* mutation was one of the potentially genetic backgrounds related to SCD and MVT. The MVT originating from the inflow-free wall and outflow tract of the right ventricle was successfully ablated using an epicardial–endocardial approach.

### *DSG2* as a pathogenic gene

ARVC/D is characterized by progressive fibro-fatty replacement and cardiacmyocyte disruption in the right or/and left ventricles. ARVC/D is also an important risk factor for SCD resulting from MVT and/or irreversible heart failure in young people [13, 14]. DSG2 is an important component of the desmosome in the cardiac intercalated disc, which interacts with desmocollin-2 through extracellular domains [15]. It has been reported that *DSG2* mutations are related to ARVC/D, hypertrophy and DCM. According to previous studies, the *DSG2 p.N271S* mutation in young transgenic mice (similar to the *N266S* mutation in humans) was associated with SCD induced by spontaneous VT, biventricular dilation and aneurysms, myocardial necrosis and heart failure [16]. According to previous report about a Chinese Han family with ARVC/D from our cardiac center, [17] we have identified a homozygous mutation of *DSG2 p.F531C* by the whole genome sequencing. The carriers of the homozygous genotype were shown to be affected with biventricular dilation and non-compaction, aneurysms in the left ventricle and spontaneous multifocal VTs. A myocardial biopsy showed interrupted, atrophic and disarranged myocardial fibers; interstitial fibers, hyperplastic, infiltrated and collagen-invaded fibers and adipocytes; and widened and destroyed intercalated discs. Some carriers with heterozygous mutation of *DSG2 p.F531C* showed incomplete penetrance [17]. Similar phenotype of ARVC/D induced by homozygous mutation of *DSG2 p.F531C* was also found in another Chinese Han family and *DSG2-F531C* knock-in mice with decreased expression of CX43. The different phenotype and its penetrance between homozygous and heterozygous of *DSG2 p.F531C* suggested the gene-dose dependent pathogenesis [18]. In this family, the patients (II:2, III:1 and II:4) with heterozygous *DSG2 p.F531C* had worse phenotypes characterized as SCD, ARVC/D, MVT and DCM. Whereas the members (II:5 and II:6) without *DSG2 p.F531C* showed delayed enhancement localized in the epicardial base or adipose infiltration in the apex of left ventricle with normal ventricular diameter, motion and function. There was no significant or incomplete cosegregation between genotype and phenotype in this family. The reasons may be as follows: Firstly, due to incomplete penetrance or delayed onset, III:2 who carried with heterozygous *DSG2 p.F531C* and detected with normal CMRI and ECG had no clinical syndrome or significant phenotype yet. Secondly, the early and abnormal cardiac changes of II:5 and II:6 without *DSG2 p.F531C* suggested that another novel genetic background not discovered or unknown may be harbored and affect the phenotypes, or even aggravate the phenotypes together with *DSG2 p.F531C*. Whether this kind of unknown genetic background existing in II:2, II:4, III:1 and III:2 or not would potentially affect the phenotypes of ARVC/D, MVT, DCM, even the occurrence of SCD. However, it was hard to draw a conclusion now.

### *KCNE5* stop-gain mutation and the risk of adverse events

We also identified a stop-gain mutation, heterozygous *KCNE5 p.D92E/E93X*. The *KCNE5* gene is located on the X chromosome (q23), consists of one exon and is highly expressed in cardiac muscle. KCNE5 modifies the function of most of the KCNQ family, Kv2.1 and Kv4.3. *KCNE5* mutations or polymorphisms predispose to Brugada syndrome, idiopathic ventricular fibrillation, subclinical QT prolongation and atrial fibrillation [19]. A male patient with Brugada syndrome and a family history of SCD carried a heterozygous mutation of *KCNE5 p.D92E/E93X*. The current densities of I_to_ were significantly increased in CHO cells transfected with *KCNE5 p.D92E/E93X* alone when compared to cells transfected with wild-type *KCNE5* or both (*KCNE5 p.D92E/E93X* and wild-type *KCNE5*). The heterozygous *KCNE5 p.D92E/E93X* mutation was inherited from the patient’s mother and heredity to his two daughters, although these three family members did not show a Brugada pattern on their ECGs or have adverse events. Interestingly, the SCD of this family was predominantly seen in the proband’s paternal side, but not his maternal side. It was speculated that *KCNE5 p.D92E/E93X* was a genetic modifier, suggesting that the causal genetic mutation for SCD had not been found. The results in that study also suggested that carrying the homozygous *KCNE5 p.D92E/E93X* mutation may influence the current density of I_to_, but carrying the mutation in the heterozygous state may not [20]. According to the population data on East Asians in the 1000 Genomes Project, the minor allele frequency of *KCNE5 p.D92E/E93X* is 0.0026 in the general population, which may not be low enough to cause a rare dominantly inherited cardiac disease.

In this family, *DSG2 p.F531C* may be one of the genetic causes of ARVC/D and MVT, as it has been seen in three Chinese Han families. The family member II:4 (male, 47 years old) also carried the *DSG2 p.F531C* mutation and had DCM, but did not have adverse events associated with MVT and SCD. ARVC/D itself is a strong risk factor for MVT and SCD. Nevertheless, there were different phenotypes and prognoses among the carriers. For example, due to incomplete penetrance, delayed onset or lack of another harboring genetic background unknown, III:2 with *DSG2 p.F531C* had no significant phenotype of ARVC/D and MVT. So it is difficult to determine the contribution of the heterozygous *KCNE5 p.D92E/E93X* mutation on the prognosis of II:2 and III:1. As compared to II:4, II:2 and III:1 were compound and heterozygous carriers of *DSG2 p.F531C* and *KCNE5 p.D92E/E93X*, which occurred with SCD in one patient and MVT in another, suggesting that the latter mutation may increase the susceptibility of SCD or MVT in this family. Therefore, although we cannot determine the exact contribution of *KCNE5 p.D92E/E93X* on SCD or MVT in this family, we can speculate that *DSG2* harbors the potentially pathogenic mutation related to cardiac structure and was the main cause of SCD and MVT. On the other hand, the heterozygous mutation of *KCNE5 p.D92E/E93X* may partly play an important role as a risk modifier. In contrast to the study mentioned above, except for the heterozygous *KCNE5 p.D92E/E93X* mutation, we found a potentially malignant genetic background of *DSG2 p.F531C* mutation expressed in the intercalated discs of cardiac structure and related to SCD and MVT.

### Catheter ablation therapy

Epicardial focus is common in approximately 41% of ARVC/D patients, due to the high probability of midmyocardial or subepicardial scars. Compared to an endocardial strategy, an epicardial–endocardial approach for ARVC/D patients may improve freedom from MVT with long-term efficacy. Acute procedural success with non-inducibility is significantly associated with long-term VT freedom [21]. The epicardial mapping and ablation are considered, when the characteristics of a patient are satisfied with the criteria as follows: the epicardial VT exit site suggests by an ECG; prior unsuccessful endocardial ablation; sub-epicardial or mid-myocardial scars shown by cardiac imaging and likelihood of epicardial circuit for underlying substrate. The ECG criteria suggesting epicardial VT exit included pseudo-delta > 34 milliseconds (ms), intrinsicoid deflection time (V_2_ lead) > 85 ms, shortest RS complex > 121 ms, and QRS duration > 211 ms [22]. For III:1 in our study, after failed ablation in the endocardium, the ICD was implanted. The ICD electrical storms recurred repeatedly. Under the guidance of the CARTO system in the second ablation, there was no low-voltage region or scar in the endocardium of both ventricles. Epicardial substrate mapping was performed, which revealed clear scars fulfilled with double, fragmented and late potentials, suggesting narrow conduction channels around the tricuspid annulus in the inferior-lateral and inferior-apical walls and the outflow tract of the right ventricle. These electrical abnormalities were more significant and severe than those of ECG and structure scanned by CT. Linear and lamellar ablation was successfully performed in these epicardial channels. After identifying potential targets with late potentials and ECGs similar to clinical MVT by endocardial pacing mapping in the median septum of the right ventricle, the endocardial ablation was performed in this region. No MVT or electrical storms recurred during one year of follow-up of III:1.

### Study limitations

In this study, although the *DSG2 p.F531C* mutation is one of the potentially pathogenic risks associated with various phenotypes of ARVC/D, MVT, SCD and DCM, the pathogenic mechanism is unknown. The novel and harboring genetic background inducing early change of ARVC/D in II:5 and II:6 is unknown and needs more detailed pedigree for analysis. The influence of heterozygous *KCNE5 p.D92E/E93X* on the prognosis of ARVC/D still needs further evaluation in vitro experiment.

### Conclusions

In a Chinese family with ARVC/D, MVT, SCD and DCM cases, we identified potential pathogenic mutations in *DSG2* and *KCNE5* by WES in a patient with ARVC/D, MVT and family history of SCD. The *DSG2 p.F531C* mutation may be an important pathogenic mutation for MVT and SCD. MVT originated from the inflow-free wall and the outflow tract of the right ventricle. The strategy of epicardial–endocardial ablation across/in the substrate scars characterized as late, fragmented or double potentials was successfully performed under the guidance of a three-dimensional mapping system, with valid prognosis during long-term follow-up. Although it is difficult to clarify the contribution of heterozygous mutation of *KCNE5 p.D92E/E93X* on MVT and SCD in this ARVC/D family, our research indicated that at least MVT and SCD occurred in some members carrying with heterozygous *DSG2 p.F531C* as potentially pathogenic background, and *KCNE5 p.D92E/E93X* as a risk modifier simultaneously.

## Additional file

Additional file 1: Predisposing genes. (DOCX 19 kb)

## Abbreviations

ARVC/D: Arrhythmogenic right ventricular cardiomyopathy/dysplasia
BWA: The Burrows-Wheeler Aligner
CMRI: Cardiac magnetic resonance imaging
CT: Cardiac computed tomography
DCM: Dilated cardiomyopathy
DSG2: Desmoglein-2
ECGs: Electrocardiograms
ICD: Implantable cardioverter-defibrillator
InDels: Insertion-deletions
MRI: Magnetic resonance image
MVT: Malignant ventricular tachycardia
SAM tools: Sequence Alignment/Map tools
SCD: Sudden cardiac death
SCD: Sudden cardiac death
SNPs: Single-nucleotide polymorphisms
WES: Whole exome sequencing
