# Supplementary material for: Case report of familial sudden cardiac death caused by a DSG2 p.F531C mutation as genetic background when carrying with heterozygous KCNE5 p.D92E/E93X mutation
Source: BMC Med Genet. 2018 Aug 21;19:148. doi: 10.1186/s12881-018-0580-2 (PMC6102856; doi:10.1186/s12881-018-0580-2)
Supplement: Supplementary file 1 — Predisposing genes. (DOCX 19 kb) [file 12881_2018_580_MOESM1_ESM.docx]

**Predisposing genes**

| TNNT2, MYH7, PLN,TCAP,ABCC9,LMNA,DMD, TAZ, TTN, VCL, EYA4, EMD, ACTN2, SGCD, ACTC1, TNNI3, TPM1, MYBPC3, LDB3, MYH6, CTF1, DES, RBM20, NEBL, TBX5, BAG3, NCOA6, ANKRD1, CSRP3, DYS, DNAJC19, DSG2, DSP, FHL2, FKTN, FOXD4, LAMP2, MYPN, NEXN, PSEN1, PSEN2, SCN5A, SDHA, SYNE1, TMPO, TNNC1, TTR, CASQ2, DTNA, GLA, GATA6, FLNC ACTC, MYL2, MYL3, PRKAG2, SERCA2a, RyR2, JPH2, DSC, TMEM43, PKP2, JUP, TGFβ3, LIM, CTNNA3, PKP4, STRN, DMPK, AMPD1, PMP22, LMX1B, FKBP12, CSX, PRDM16, ZASP1, ND1, NKX2.5, NOTCH1, MCTP2, FOXP1, PROX1, HAND1, GJA1, RAF1, FOXC2, FOXL1, CTNI, OS1AP, SCN10A, CX43, CXADR, BAZ2B, ADRB2, GLN27, MT-RNR1，MT-TV，MT-TL1，MT-ND1，MT-TI，MT-TW，MT-CO2，MT-TK，MT-ATP6，MT-TG，MT-ND4，MT-TH，MT-TL2，MT-ND5，MT-CYB，MT-DLOOP，COX9，COX10，COX15，SCO2，SLC25A4，SLC25A3，AARS2，TSFM, KCNQ1, KCNH2, ANK2, KCNE1, KCNE2, KCNJ2, KCNJ5, CACNA1C, CAV3, SCN4B, AKAP9, SNTA1, S1103Y, CALM1, CALM2, CACNB2, GPD1L, SCN1B, KCNE3, SCN3B, RANRF, SLMAP, SCN2B, CACNB2b, CACNA2D1, KCNJ8, KCNE5, KCND3, KCND2, HCN4, TRMP4, FGF12, PXDNL, CLASP2, IRX5, DPPX, HEY2, MOG1, TRDN, ANKB, KCNA5, NPPA, GJA5, NUP155, MYL4 |
| --- |
| Note: these genes predisposing to cardiomyopathies and arrhythmias, including dilated cardiomyopathy, hypertrophic cardiomyopathy, arrhythmogenic cardiomyopathy/dysplasia, Non-compaction of ventricular myocardium, congenital ventricular dysplasia, restrictive cardiomyopathy, sudden death, acquired risks of sudden cardiac death, mitochondrial cardiomyopathy, long QT syndrome, Brugada syndrome, catecholaminergic polymorphic ventricular tachycardia, sick sinus syndrome, pre-excitation syndrome, cardiac conduction disease, short QT syndrome, and atrial fibrillation. Some pathogenic mutations of these genes have been reported in previous studies. |
